# Children and caregiver proxy quality of life from peanut oral immunotherapy trials

**DOI:** 10.1002/clt2.12213

**Published:** 2022-12-19

**Authors:** Audrey Dunn Galvin, Andrea Vereda, Pablo Rodríguez del Río, Antonella Muraro, Carla Jones, Robert Ryan, David Norval, Jennifer Jobrack, Aikaterini Anagnostou, Julie Wang

**Affiliations:** ^1^ University College Cork Cork Ireland; ^2^ Aimmune Therapeutics, a Nestlé Health Science Company London UK; ^3^ Infantil Niño Jesús University Paediatric Hospital Madrid Spain; ^4^ Food Allergy Centre Padua University Hospital Padua Italy; ^5^ Allergy UK Sidcup Kent UK; ^6^ Food Allergy Pros, LLC Chicago Illinois USA; ^7^ Texas Children's Hospital & Baylor College of Medicine Houston Texas USA; ^8^ Icahn School of Medicine at Mount Sinai New York New York USA

**Keywords:** caregiver proxy, health‐related quality of life, oral immunotherapy, peanut allergy

## Abstract

**Background:**

Health‐related quality of life (HRQoL) is significantly and substantially reduced in individuals with peanut allergy due to many factors associated with unanticipated or potentially fatal reactions. Further insight on the impact of peanut oral immunotherapy in managing peanut allergy on HRQoL is needed. The aim of this analysis was to assess effects of peanut (*Arachis hypogaea*) allergen powder‐dnfp (PTAH), a biologic drug for peanut oral immunotherapy, on HRQoL from three phase 3 and two follow‐on trials of PTAH.

**Methods:**

HRQoL assessments from participants aged 4–17 in the PALISADE (ARC003), ARC004 (PALISADE follow‐on), ARTEMIS (ARC010), RAMSES (ARC007), and ARC011 (RAMSES follow‐on) trials were included in this analysis. Responses on the Food Allergy Quality of Life Questionnaire (FAQLQ) and Food Allergy Independent Measure (FAIM) were evaluated by age group and respondent (self or caregiver proxy). Data were analyzed with descriptive statistics and Student *t* tests.

**Results:**

Baseline FAQLQ and FAIM total scores appeared comparable between PTAH‐ and placebo‐treated participants. Self and caregiver proxy‐reported total scores on the FAQLQ for PTAH‐treated participants generally improved at trial exit versus baseline; FAIM total scores improved throughout all trials. The tendency for improvement in FAQLQ total scores from baseline for PTAH appeared larger in self versus caregiver proxy‐reports. Between treatment groups, PTAH was generally favored in the PALISADE and ARTEMIS trials; differences varied in the RAMSES trial based on age and respondent types.

**Conclusions:**

PTAH for the management of peanut allergy in children appeared to have a beneficial effect on HRQoL in trials. Improvements were seen despite rigors of trial participation.

## 1 INTRODUCTION

Health‐related quality of life (HRQoL) is significantly reduced in patients with food allergy and their families.^1^, ^2^, ^3^, ^4^ The impact of food allergy extends beyond clinical outcomes such as number or severity of reactions to encompass fear/anxiety associated with unanticipated/potentially fatal reactions.^1^, ^2^, ^3^, ^4^ Additionally, food allergy imposes restrictions and limitations on social activities and increases risk of depression or anxiety.^5^ Understanding the impact of food allergy on HRQoL can guide policy making and healthcare resource allocation.^6^, ^7^

Peanut (*Arachis hypogaea*) allergen powder‐dnfp (PTAH; previously AR101; Palforzia^®^) is a biologic drug for peanut oral immunotherapy (OIT) approved in the United States and Europe to mitigate allergic reactions following accidental exposure to peanut in individuals aged 4–17 years with a confirmed diagnosis of peanut allergy.^8^, ^9^ PTAH demonstrated efficacy and safety in phase 2 and 3 trials.^10^, ^11^, ^12^, ^13^ Recently published guidelines for food allergy management acknowledge peanut OIT may induce desensitization in children allergic to peanut and support the use of a licensed/regulatory approved preparation for those aged 4–17 years with a confirmed diagnosis.^14^, ^15^

A systematic review of parent‐reported measures of the psychosocial impact of food allergy on patients and their families found that practical, relevant, reliable, and valid parent‐reported measures remain an unmet need.^16^ Working with patients and their families and determining how support can be best provided requires a flexible approach. Regular follow‐up is key to addressing changing psychosocial/dietary needs, monitoring allergy resolution, and evaluating treatment response. Additionally, comprehensive multidisciplinary or multiprofessional teams (including access to support from dietitians, psychologists, pharmacists, patient/caregiver/advocate organizations, and the food industry) are needed to optimize quality of life for individuals in complex situations.^17^, ^18^ Studies to date assessing HRQoL change following OIT demonstrate encouraging but mixed results. Whereas individual clinical and real‐world studies have reported improvements with peanut OIT,^11^, ^13^, ^19^, ^20^, ^21^, ^22^, ^23^ a meta‐analysis found no difference in HRQoL scores between OIT and placebo,^24^ and a small study found parents, but not children, reported improvement.^25^ Notably, providing psychological support to patients undergoing OIT and their families may improve HRQoL.^26^

While several HRQoL instruments for various age groups and respondents (self vs. parent) were found to be reliable, valid, and responsive, evidence for sensitivity to change in response to treatment was limited, as was the determination of a clinical minimal important difference (MID).^27^ The Food Allergy Quality of Life Questionnaire has undergone the most thorough validation process with psychometric property testing; the Food Allergy Independent Measure (FAIM) can be used concurrently as a correlating measure.^28^ Previously, HRQoL data from ARTEMIS^11^ demonstrated clinically meaningful improvements in PTAH‐treated versus placebo‐treated participants, and ARC004^13^ showed clinically meaningful improvements in PTAH‐treated participants, usually increasing with longer treatment duration on the FAQLQ and FAIM. The objective of this analysis was to assess effects of PTAH on HRQoL from multiple trials, including longer‐term treatment and a larger population.

## 2 METHODS

### 2.1 Trial design and participants

HRQoL assessments from PALISADE (NCT02635776), ARC004 (NCT02993107), ARTEMIS (NCT03201003), RAMSES (NCT03126227), and ARC011 (NCT03337542) were included in this analysis. HRQoL assessment was a prespecified endpoint in all trials. For ARC004, only participants receiving PTAH daily (the approved dosage for maintenance treatment, 300 mg daily)^8^, ^9^ were included (cohorts 1 and 3A). Details of PALISADE,^10^ ARC004,^12^ and ARTEMIS^11^ have previously been reported. Participants included in this analysis were aged 4–17 years at trial enrollment. Trials were approved by institutional review boards and informed consent/assent was obtained from participants before participation. Additional trial information is available in Table 1.

**TABLE 1:** PTAH phase 3 trials included in the HRQoL analysis

	ARC003 (PALISADE^a^)	ARC004	ARC010 (ARTEMIS^b^)	ARC007 (RAMSES^c^)	ARC011
Trial design	Randomized, double‐blind, placebo‐controlled study	Open‐label, ARC003 follow‐on, safety study	Randomized, double‐blind, placebo‐controlled study	Randomized, double‐ blind, placebo‐controlled safety study	Open‐label, ARC007 follow‐on, safety study
Geographic region	North America; Europe	North America; Europe	Europe	North America	North America
Participants^d^	*n* = 496	*n* = 351 (cohort 1, *n* = 109; cohort 3A, *n* = 31)^e^	*n* = 175	*n* = 506	*n* = 242
Treatment duration	∼12 months	∼6 (cohort 1) or 12 months (cohort 3A)	∼9 months	∼6 months	∼6 months
Key eligibility criteria	Clinical history of allergy to peanut or peanut‐containing foods, psIgE ≥0.35 kUA/L, mean peanut SPT wheal diameter ≥3 mm compared with negative saline control, DLS with ≤100 mg peanut protein on screening DBPCFC	Received PTAH and completed ARC003 (PALISADE)	Clinical history of allergy to peanut or peanut‐containing foods, psIgE ≥0.35 kUA/L, mean peanut SPT wheal diameter ≥3 mm compared with negative saline control, DLS with ≤300 mg peanut protein on screening DBPCFC	History of physician‐diagnosed IgE‐mediated peanut allergy including onset of characteristic allergic signs and symptoms within 2 h of known oral exposure to peanut or peanut‐containing foods, psIgE ≥14 kUA/L, mean peanut SPT wheal diameter ≥8 mm compared with negative saline control	Received PTAH and completed ARC007 (RAMSES)

Abbreviations: DBPCFC, double‐blind, placebo‐controlled food challenge; DLS, dose‐limiting symptoms; HRQoL, health‐related quality of life; psIgE, peanut‐specific immunoglobulin E; PTAH, peanut (*Arachis hypogaea*) allergen powder‐dnfp; SPT, skin prick test.

^a^ Peanut Allergy Oral Immunotherapy Study of AR101 for Desensitization in Children and Adults.

^b^ AR101 Trial in Europe Measuring Oral Immunotherapy Success in Peanut Allergic Children.

^c^ Real‐World AR101 Market‐Supporting Experience Study in Peanut‐Allergic Children Ages 4–17 Years.

^d^ Safety population sample size; only includes participants aged 4–17 years.

^e^ Cohort 1 included enrolled patients taking PTAH 300 mg daily for 28 weeks; cohort 3A included randomized patients taking PTAH 300 mg daily for 56 weeks.

### 2.2 Assessments and endpoints

HRQoL assessments were conducted using the FAQLQ^29^, ^30^, ^31^ and FAIM.^32^ Items and domains on versions vary depending on the respondent (self or caregiver proxy) and age group of the participant. FAQLQ examines the effect of restrictions and psychosocial effects caused by food allergy. The number of items/domains varies by version, ranging from 23 to 30 questions. Domains for various versions could vary slightly depending upon age and include allergy avoidance/dietary restrictions, social/dietary limitations, emotional impact, risk of accidental exposure, or food anxiety. FAQLQ was scored on a 7‐point scale from 1, indicating minimal impairment (or no impact), to 7, indicating maximal impairment (or extreme impact). FAIM measures perception of disease severity and assesses future expectations associated with accidental ingestion of an allergen by the individual with food allergy. The instrument consists of six questions: four relating to expectation of outcome and two relating to disease severity. The caregiver proxy version includes questions relating to perception of disease severity and expectation of allergen exposure outcome consisting of eight questions for participants aged 4–12 years and four questions for participants aged 13–17 years. Items include the chance of having a severe reaction, dying from accidental exposure, dealing with accidental exposure, or the perceived amount of foods which must be avoided. FAIM was scored on a 7‐point scale with 1 indicating limited perception of severity and 7 indicating greatest perception of severity.

**FIGURE 1:**
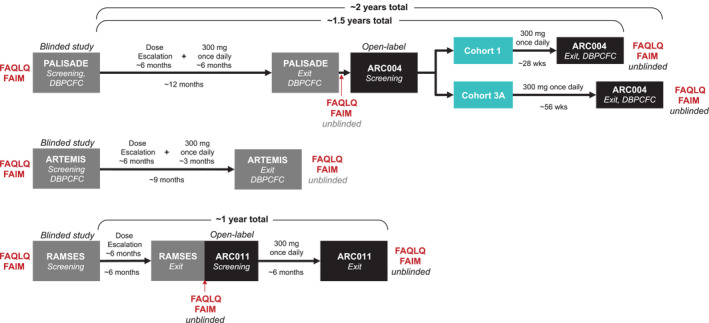
Study design. DBPCFC, double‐blind, placebo‐controlled food challenge; FAIM, Food Allergy Independent Measure; FAQLQ, Food Allergy Quality of Life Questionnaire.

The FAQLQ and FAIM were administered at prespecified time points in each study (Figure 1). In PALISADE and ARTEMIS, HRQoL instruments were completed at screening/baseline before the double‐blind, placebo‐controlled food challenge (DBPCFC) and after completion of the exit DBPCFC and unblinding, which happened immediately. In ARC004 (PALISADE open‐label extension), HRQoL instruments were completed at screening/baseline prior to the start of treatment (concurrent with PALISADE exit procedures) and after completion of the exit DBPCFC. In RAMSES, HRQoL instruments were completed at screening/baseline prior to randomization (no food challenges were conducted in RAMSES) and after the completion of exit procedures and unblinding. In ARC011 (RAMSES open‐label extension), HRQoL instruments were completed prior to the start of treatment (ie, completed at screening/baseline if not completed at the RAMSES exit visit) and the ARC011 exit visit. FAQLQ and FAIM were completed by participants aged 8–17 years and caregiver proxies of all participants aged 4–17 years. Participants completed the same HRQoL instrument version at baseline and exit, even if their age at trial exit qualified them to receive a different HRQoL version (ie, the version for the next age range if the child was a year older at study end), so baseline and exit scores could be compared. A MID of 0.5 was used as a threshold.^33^ Additional detail on scoring is found in the Supporting Information.

### 2.3 Statistical analyses

Scores were summarized with descriptive statistics. To compare treatment and placebo group scores, a Student *t* test (paired or unpaired as appropriate) was used. Mean change was reported for within‐group (PTAH) and between‐group (PTAH vs. placebo) changes in scores from screening to exit visit. A change in score of ≥0.5 indicated an improvement of quality of life that reached or exceeded the MID. Statistical analyses were performed using Statistical Analysis Software, version 9.4 (SAS Institute, Inc.).

## 3 RESULTS

### 3.1 Descriptive analysis: Individual trials

#### 3.1.1 PALISADE and ARC004

In PALISADE, baseline total FAQLQ and FAIM scores appeared comparable between PTAH‐treated and placebo‐treated participants (Table 2). In PALISADE and ARC004, FAQLQ caregiver proxy‐report scores of teenagers and self‐report were higher on average than for caregiver proxy‐report of younger children, regardless of treatment group. In PALISADE, FAIM scores for caregiver proxy‐report were higher versus self‐report.

**TABLE 2:** FAQLQ and FAIM baseline scores

*n*; total mean score (SD)	PALISADE		ARC004^a^		ARTEMIS		RAMSES		ARC011
	PTAH	PBO	PTAH (1)	PTAH (3A)	PTAH	PBO	PTAH	PBO	PTAH
FAQLQ total mean scores at baseline									
Caregiver proxy‐report									
4–6 years	84	22	10	6	43	13	78	40	58
	3.79 (1.50)	3.48 (1.50)	3.34 (1.41)	3.54 (1.39)	3.29 (1.49)	3.60 (1.29)	3.65 (1.59)	3.41 (1.44)	3.78 (1.60)
7–12 years	165	65	53	13	58	18	171	88	127
	3.89 (1.29)	4.24 (1.40)	3.95 (1.40)	3.81 (1.53)	3.53 (1.60)	3.35 (1.10)	4.04 (1.42)	3.94 (1.25)	4.10 (1.42)
13–17 years	79	27	24	8	27	12	84	37	54
	4.56 (1.20)	4.11 (1.22)	4.81 (1.18)	4.52 (1.07)	4.50 (1.00)	4.51 (1.15)	4.36 (1.14)	4.56 (1.16)	4.34 (1.18)
Self‐report									
8–12 years	146	56	32	9	43	15	134	71	99
	4.22 (1.30)	4.40 (1.41)	4.48 (1.28)	3.59 (1.22)	4.06 (1.24)	4.03 (1.61)	4.27 (1.41)	4.26 (1.29)	4.42 (1.41)
13–17 years	86	29	25	8	28	12	86	37	55
	4.66 (1.17)	4.41 (1.04)	4.54 (1.14)	5.10 (1.17)	4.84 (1.21)	4.50 (1.23)	4.39 (1.19)	4.66 (1.22)	4.23 (1.25)
FAIM total mean scores at baseline									
Caregiver proxy‐report									
4–12 years	267	90	68	21	88	30	227	119	171
	4.43 (0.91)	4.52 (0.94)	4.21 (1.01)	4.60 (0.89)	4.17 (1.07)	4.43 (0.89)	4.52 (1.03)	4.36 (1.03)	4.56 (0.99)
13–17 years	93	29	27	8	26	12	80	35	55
	4.70 (1.03)	4.92 (0.89)	5.13 (0.88)	4.53 (0.73)	4.80 (0.96)	4.44 (0.93)	4.80 (1.02)	5.02 (0.89)	3.63 (1.04)
Self‐report									
8–12 years	157	58	34	10	39	15	132	71	98
	3.59 (1.01)	3.68 (0.97)	3.62 (0.90)	3.55 (0.64)	3.44 (0.82)	3.40 (0.77)	3.81 (1.09)	3.67 (0.94)	3.91 (1.17)
13–17 years	95	29	27	8	26	12	86	34	55
	3.72 (0.88)	3.83 (0.83)	3.78 (0.93)	3.98 (0.61)	4.00 (1.06)	3.28 (1.11)	3.73 (1.02)	3.80 (0.90)	3.63 (1.04)

*Note*: Both FAQLQ and FAIM use a 7‐point scale; 1 indicates minimal impairment and 7 indicates maximal impairment.

Abbreviations: FAIM, Food Allergy Independent Measure; FAQLQ, Food Allergy Quality of Life Questionnaire; PBO, placebo; PTAH, peanut (*Arachis hypogaea*) allergen powder‐dnfp.

^a^ Baseline scores for ARC004 were baseline FAQLQ and FAIM scores for entry into PALISADE.

**FIGURE 2:**
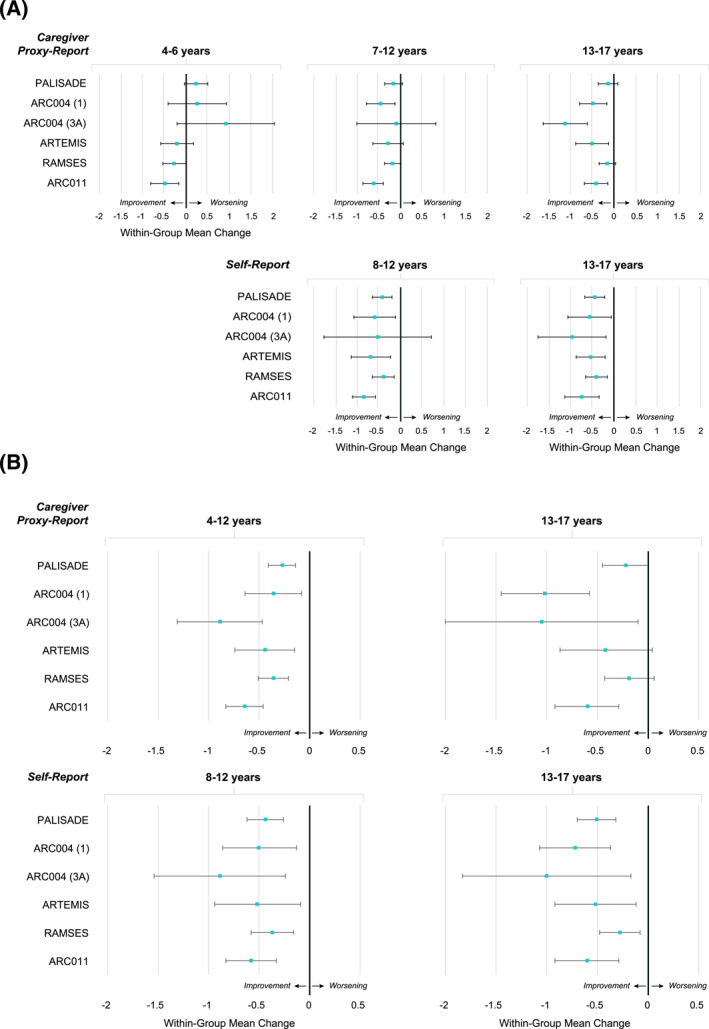
Change in total scores from baseline for PTAH by trial. (A) FAQLQ change in total scores from baseline for PTAH by trial. (B) FAIM change in total scores from baseline for PTAH by trial. Data shown represent within‐group mean change (95% confidence interval). Negative change indicates improvement. ARC004 (1) and ARC004 (3A) represent cohort 1 and cohort 3A, respectively, in the ARC004 trial. FAIM, Food Allergy Independent Measure; FAQLQ, Food Allergy Quality of Life Questionnaire; PTAH, peanut (*Arachis hypogaea*) allergen powder‐dnfp.

In PALISADE, FAQLQ total score improvement from baseline in PTAH‐treated participants was observed globally (ie, across all age groups and respondents), except for caregiver proxy‐report of children aged 4–6 years (Figure 2A). In PTAH‐treated participants, improvement in FAIM total scores from baseline was observed globally (Figure 2B).

The proportion of participants in PALISADE with MID in FAQLQ total scores was numerically higher for PTAH‐treated versus placebo‐treated participants globally, except for self‐report by children aged 8–12 years; the MID in FAIM total scores was higher on average across age groups and respondents. In ARC004, the findings for MID in FAQLQ and FAIM total scores appeared variable among age groups and respondents in each cohort.

**FIGURE 3:**
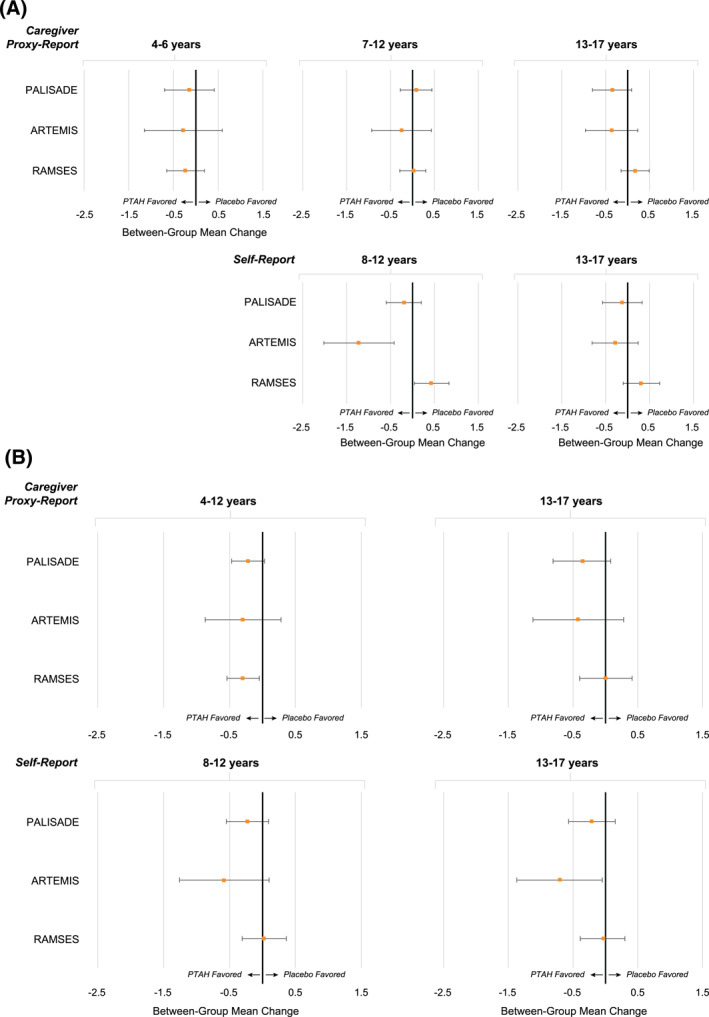
Differences in total scores between treatment groups. (A) Differences in FAQLQ total scores between treatment groups. (B) Differences in FAIM total scores between treatment groups. Data shown represent between‐group mean change (95% confidence interval). Negative difference indicates PTAH is favored. Comparisons are not available for ARC004 or ARC011 as these were open‐label, follow‐on trials, with no placebo arm. FAIM, Food Allergy Independent Measure; FAQLQ, Food Allergy Quality of Life Questionnaire; PTAH, peanut (*Arachis hypogaea*) allergen powder‐dnfp.

Comparisons between PTAH‐treated and placebo‐treated participants on FAQLQ and FAIM total scores show point estimates favoring PTAH; however, 95% confidence intervals were wide (Figure 3A,B).

#### 3.1.2 ARTEMIS

Baseline total scores for FAQLQ and FAIM followed the same pattern as described above for PALISADE and ARC004 (Table 2).

Improvement in FAQLQ and FAIM total scores from baseline in PTAH‐treated participants was observed globally (Figure 2A,B).

Proportions of participants who met or exceeded MID for FAQLQ total scores were higher for PTAH‐treated than placebo‐treated participants globally, except for the caregiver proxy‐report for children aged 13–17 years (lower) and 4–6 years (slightly higher). Among self‐report for age groups 8–12 and 13–17 years, proportions of PTAH‐treated participants with MID were ∼30 percentage points higher than for placebo‐treated participants. The proportion who met or exceeded MID for FAIM total scores trended toward PTAH versus placebo.

Globally for FAQLQ total scores, PTAH differed compared with placebo with this difference most robust for self‐report by children aged 8–12 years (Figure 3A). For FAIM total scores, PTAH was different compared with placebo globally, the difference appearing most robust for self‐report by children aged 13–17 years (Figure 3B).

#### 3.1.3 RAMSES and ARC011

In RAMSES, baseline FAQLQ and FAIM total scores were comparable in PTAH‐treated and placebo‐treated participants in each age group and by respondent (Table 2). In both RAMSES and ARC011, FAQLQ scores for the caregiver proxy‐report of teenagers and self‐report trended higher versus caregiver proxy‐report of younger children. FAIM scores for the caregiver proxy‐report were slightly higher than for self‐report in RAMSES and ARC011.

In RAMSES, FAQLQ and FAIM total score improvement in PTAH‐treated participants was observed globally (Figure 2A,B). In ARC011, FAQLQ and FAIM total score improvement was observed globally and the magnitude of improvement generally appeared greater than in RAMSES.

In RAMSES, proportions of participants with MID in FAQLQ total scores appeared higher for placebo‐treated versus PTAH‐treated participants. In ARC011, ∼40% or more of participants had MID in FAQLQ and FAIM total scores. In RAMSES, proportions of participants with MID in FAIM total scores appeared variable.

For FAQLQ total scores, the difference between treatment groups appeared to favor placebo among self‐reports (Figure 3A). Results for FAIM total scores had wide 95% confidence intervals (Figure 3B).

### 3.2 Descriptive analysis: Comparisons across trials

Across parent trials (PALISADE, ARTEMIS, and RAMSES), history of prior reaction to peanut and concurrent asthma or other food allergy were broadly comparable; sensitivity to peanut appeared higher in the RAMSES trial (Table S1). Across all trials, baseline FAQLQ total scores generally appeared similar. Caregiver proxies responding for teenagers appeared to have higher mean scores than for children aged 4–6 years; children seemed to have higher mean scores than for caregiver proxies responding for them. Self‐ and caregiver proxy‐reported FAQLQ total scores showed trends toward greater improvements in those who received PTAH for at least 9 months versus placebo (PALISADE and ARTEMIS). After ∼6 months of PTAH treatment (RAMSES), this trend toward improvement was only observed in caregiver proxy‐reported FAQLQ total scores for the youngest participants aged 4–6 years.

Regarding change in FAQLQ total scores from baseline for active treatment, a tendency toward improvement (ie, reduction) in scores was observed and appeared larger in self‐reports. Self‐reported FAQLQ total scores for participants aged 8–12 years and 13–17 years showed similar and consistent improvements with up to 2 years of treatment. FAQLQ domains demonstrating a higher level of improvement for PTAH treatment were “emotional impact” for caregiver proxies for children 13–17 years and “allergy avoidance and dietary restrictions” and “risk of accidental exposure” for children 8–12 years in the ARTEMIS trial. Curiously, placebo showed more improvement on “risk of accidental exposure” in the RAMSES trial (Table S2).

The greatest improvements in FAIM total scores were seen in PTAH‐treated participants aged 13–17 years. Domains favoring PTAH over placebo in randomized, controlled trials pertained to the risk of severe reaction and death (Table S3). In PALISADE, ARTEMIS, RAMSES, and ARC011 trials, HRQoL based on FAQLQ and FAIM total scores generally improved in participants receiving PTAH. Proportion of participants with an improvement of FAQLQ and FAIM total scores reaching the MID varied across trials; this proportion of participants was higher for FAQLQ self‐report versus caregiver proxy‐report and appeared similar across self and caregiver proxy‐report for FAIM. Compared with placebo, differences in FAQLQ and FAIM total scores generally favored PTAH; results appeared particularly robust for self‐report in ARTEMIS compared with other placebo‐controlled trials.

## 4 DISCUSSION

Children entering PTAH trials evaluated in this analysis had impaired HRQoL at baseline/trial enrollment. Based on FAQLQ and FAIM total scores, PTAH‐treated participants generally reported improvements (particularly for domains relating to allergy avoidance/dietary restrictions and risk of accidental exposure on the FAQLQ and death and severe reaction on the FAIM) in HRQoL over time; this improvement was particularly noticeable in ARC011, which included participants electing to continue a PTAH trial for an additional 6 months. Participants with worse HRQoL when entering the trial tended to improve the most. Across trials, proportions of participants experiencing improvement (of at least the MID) were substantial for all enrolled participants. FAIM total score change from baseline for PTAH appeared to improve regardless of differences in trial design or duration.

Improvement in HRQoL following OIT for food allergy was consistent with previous findings.^11^, ^13^, ^19^, ^20^, ^34^, ^35^ Variability in responses on the FAQLQ by age groups and respondents has also been reported previously and may be due to differences in understanding/perceptions of allergy by children or their parents.^34^, ^36^, ^37^ In a systematic review and meta‐analysis, OIT for peanut allergy did not improve HRQoL based on MID, although the level of evidence was considered low.^24^ In our analyses, improvement based on MID was noticeable for both patients receiving PTAH and those receiving placebo, which may not be surprising given the context (ie, blinded interventions for peanut allergy) and that desensitization to peanut cannot be felt during treatment (ie, only during accidental peanut exposure or during an oral food challenge). This contrasts other disease states, such as asthma,^38^, ^39^, ^40^ where improvement of signs/symptoms in real time may give the patient apparent measures of efficacy. Change in FAQLQ and FAIM total scores generally trended toward greater improvement in the open‐label extension trials compared with the parent trial, suggesting that improvements in HRQoL are consistent, sustained, and increasing with treatment duration, probably due to a gradual change in patient and parent mindsets and thus greater improvement as they realize their (or their childrens') higher level of protection. This improvement is likely to continue in the real world, following trial end.^41^

While FAQLQ and FAIM have been used to evaluate HRQoL in patients with food allergy, including those with peanut allergy, these questionnaires were not specifically developed for peanut allergy. Currently, HRQoL assessments with validation and psychometric testing in a peanut allergy population by various age cutoffs are unavailable. Yet, the FAQLQ was determined to be relevant for patients with peanut allergy.^42^ The questionnaires may be limited in their sensitivity to detect change in HRQoL related to treatment. For example, domains that may be resistant to change include those related to allergen avoidance or dietary restrictions (habits which have been adopted and reinforced over long periods of time), particularly as PTAH and other treatments are intended to prevent reactions and not intended to cure the patient of peanut allergy or support introduction of peanut‐containing foods in the diet; patients must continue to avoid peanut.^8^, ^9^ The effect size or MID threshold may be different based on age or respondent; a MID of 0.45 has previously been reported for parents of children with food allergy post food challenge.^33^ HRQoL instruments are a snapshot of the respondent's assessment at a point in time; while respondents may consider perspectives and events that have recently occurred, they may not capture changes over time or be overly impacted by acute changes. Timing of FAQLQ/FAIM assessments during trials is important and relevant to interpretation.

For participants entering a clinical trial where procedures are rigorous, precise, and closely monitored, HRQoL may be affected by fear and/or anxiety associated with screening activities, apprehension with blinded treatment, concerns with deliberate allergen exposure, or starting study treatment with initial dose escalation and continuation to updosing. Substantial improvements in HRQoL related to treatment would not be expected as participants/caregivers are blinded to treatment, so their perceived threat of anaphylaxis (and the associated psychosocial burden) likely remains constant. In unblinded studies, HRQoL improvements have been seen earlier in OIT and maintained with continued treatment.^20^, ^21^ HRQoL changes have been observed with oral food challenges regardless of challenge results,^43^, ^44^ and as DBPCFCs were conducted in PALISADE, ARC004, and ARTEMIS, the timing of the FAQLQ and FAIM in relation to food challenges could have affected responses. Although improvements in HRQoL for up to 6 months have been observed after a food challenge,^44^ HRQoL improvements were still observed in the RAMSES and ARC011 trials, neither of which conducted a DBPCFC, suggesting that improvements in HRQoL were not just related to information obtained from the food challenge. As noted previously, some domains may take more time to show change due to long‐held perceptions and behaviors.

Despite these considerations for the FAQLQ and FAIM, improvement was observed with PTAH treatment versus placebo in as little as 6 months in the RAMSES trial. In the follow‐on or extension studies (ARC004 and ARC011) wherein participants elected to receive or continue open‐label PTAH, HRQoL improvements appeared more pronounced with the exception of HRQoL per FAQLQ total scores reported by caregivers of children aged 4–6 years in ARC004, possibly due to study fatigue from the length of trial participation, which can be particularly challenging for parents of young children. Findings of improved HRQoL have also been documented in unblinded, controlled trials or after unblinding in blinded peanut immunotherapy trials.^22^, ^23^, ^45^ Thus, the results are positive because there is a change in such a short period of time and within the very strict/demanding environment of a clinical trial setting.

As reducing the psychosocial impact of food allergy has been identified as a key research priority, reliable and valid instruments and measures are needed to determine clinically meaningful outcomes associated with the treatment/management of food allergy.^28^, ^46^ The development of patient‐reported outcome measures and assessment tools is a complex science that requires testing for multiple measures including validity (particularly construct validity), reliability/reproducibility, usability, feasibility, internal consistency, responsiveness, and interpretability to ensure that outcome measures are psychometrically sound, person‐centered, meaningful, amenable to change (ie, before and after treatment), and implementable.^28^ Additionally, further study is needed in the translation of HRQoL instruments primarily used in research for use in clinical practice. Because the economic burden of peanut allergy is substantial, measurable HRQoL outcomes can help contextualize treatment benefits to allow payers to better integrate OIT into formularies and treatment continua.^2^, ^47^

Heterogeneity in trial design with regard to treatment duration, eligibility criteria, or blinding could have affected HRQoL outcomes. While PALISADE, ARTEMIS, and RAMSES were blinded trials, the FAQLQ and FAIM were assessed prior to the start of treatment and at the end of the study; knowledge of treatment allocation after unblinding may have affected responses. Fewer patients received active treatment for ∼2 years versus up to 1.5 years; thus, longer‐term results should be interpreted cautiously. However, a strength is that the overall analysis included over 1000 patients evaluated for HRQoL outcomes. Participants and their families interested in clinical trial participation may also be severely impacted in their HRQoL at baseline (ie, higher severity/frequency of reactions or exhausted available treatment options). Other factors that may affect the generalizability of the study findings to the broader population with peanut allergy include motivation for trial participation, access to investigators or study sites, and knowledge/awareness of PTAH treatment. Unfortunately, given the different study designs, an overall analysis adjusting for baseline characteristics was not possible. Analysis of patients with multiple food allergies compared with peanut allergy only were not conducted, nor were analyses to identify potential relationships between prior history of reactions, reactions on the oral food challenges, systemic allergic reactions, or epinephrine use during the trial on HRQoL assessments. Previous studies assessing the HRQoL of caregivers of children with food allergy found less improvement in the caregiver HRQoL for those with children with comorbid asthma,^48^ demonstrating that the presence of comorbidities can temper the potential HRQoL improvements associated with immunotherapy. While PTAH was generally favored based upon the FAQLQ and FAIM scores in placebo‐controlled trials, confidence intervals were broad, encompassing zero; thus, each confidence interval should be examined from a clinical perspective to assess trends to estimate the probability that observed changes in an apparent trend represent true differences in HRQoL improvements. *p*‐values for comparisons were not available due to the exploratory and post hoc nature of this analysis. Despite the limitations, results of blinded and open‐label trials may help provide a detailed picture of the impact of OIT on HRQoL.

Overall, use of PTAH in trials for the management of peanut allergy in children generally appeared to have a beneficial effect on HRQoL. Future studies may be conducted to explore the impact of baseline demographic or clinical characteristics, results of interaction testing, and discrimination ability for various questions for individuals with peanut allergy receiving PTAH.

## AUTHOR CONTRIBUTIONS

**Audrey Dunn Galvin** contributed to the design of the study protocol and statistical analysis plan; analysis and interpretation of data; drafting the manuscript and revising it critically; and approving the version to be published. She agrees to be accountable for all aspects of the work. **Andrea Vereda** contributed to the design of the study protocol; analysis and interpretation of data; reviewing the manuscript and revising it critically; and approving the version to be published. She agrees to be accountable for all aspects of the work. **Pablo Rodríguez del Río** was a primary investigator and contributed to running the trial, treating participants, and the collection, analysis, and interpretation of data; drafting the manuscript and revising it critically; and approving the version to be published. He agrees to be accountable for all aspects of the work. **Antonella Muraro** was a primary investigator and contributed to running the trial, treating participants, and collection of data; reviewing the manuscript and revising it critically; and approving the version to be published. She agrees to be accountable for all aspects of the work. **Carla Jones** contributed to the collection, analysis, and interpretation of data; drafting the manuscript and revising it critically; and approving the version to be published. She agrees to be accountable for all aspects of the work. **Robert Ryan** contributed to the design of the statistical analysis plan; analysis and interpretation of data; drafting the manuscript and revising it critically; and approving the version to be published. He agrees to be accountable for all aspects of the work. **David Norval** contributed to the design of the statistical analysis plan; analysis and interpretation of data; drafting the manuscript and revising it critically; and approving the version to be published. He agrees to be accountable for all aspects of the work. **Jennifer Jobrack** contributed to the collection, analysis, and interpretation of data; drafting the manuscript and revising it critically; and approving the version to be published. She agrees to be accountable for all aspects of the work. **Aikaterini Anagnostou** was a primary investigator and contributed to running the trial, treating participants, and the collection, analysis, and interpretation of data; drafting the manuscript and revising it critically; and approving the version to be published. She agrees to be accountable for all aspects of the work. **Julie Wang** was a primary investigator and contributed to the design of the study protocol, running the trial, and treating participants; analysis and interpretation of data; drafting the manuscript and revising it critically; and approving the version to be published. She agrees to be accountable for all aspects of the work.

## CONFLICT OF INTEREST

**Audrey Dunn Galvin** reports grants or contracts from DBV Technologies, WAO, and EAACI and payment or honoraria for lectures, presentations, speakers' bureaus, manuscript writing, or educational events by DBV Technologies, Nutritics, and Nutricia. **Andrea Vereda** is an employee of Aimmune Therapeutics, a Nestlé Health Science company. **Pablo Rodríguez del Río** reports speaker fees from Aimmune Therapeutics, a Nestlé Health Science company. **Antonella Muraro** reports speaker fees from Aimmune Therapeutics, a Nestlé Health Science company, DBV Technologies, Viatris‐Mylan, ALK, Nestlé Health Science, Nestlé Purina, Novartis, and Nutricia Research; advisory board for Novartis, Regeneron, Aimmune Therapeutics, a Nestlé Health Science company, DBV Technologies, and Viatris; and grants from Aimmune Therapeutics, a Nestlé Health Science company, and Sanofi. **Carla Jones** reports nonpersonal sponsorship for Allergy UK Primary Care Masterclass for HCPs in 2018 and 2019, as well as nonpersonal sponsorship for Allergy UK webinar for HCPs and article in Allergy Today 2021 by Aimmune Therapeutics, a Nestlé Health Science company; nonpersonal sponsorship for Allergy UK for a survey and report 2018 and nonpersonal sponsorship for Allergy UK for an abstract, poster, and round table report 2019 by DBV; fees for Allergy UK to attend European Insights Workshop October 2018 – reading survey, review program, attend workshop, reading and meeting attendance October 2019, and review of educational handbooks and videos 2020 by Aimmune Therapeutics, a Nestlé Health Science company; nonpersonal sponsorship for Allergy UK for review and dissemination of vignette study 2020 by DBV Technologies; and a colleague was supported to attend PAAM 2019 as presenting a poster by DBV Technologies. **Robert Ryan** is an employee of Aimmune Therapeutics, a Nestlé Health Science company. **David Norval** is an employee of Aimmune Therapeutics, a Nestlé Health Science company. **Jennifer Jobrack** was a previous consultant to Aimmune Therapeutics, a Nestlé Health Science company, and spoke at ACAAI in February 2020. **Aikaterini Anagnostou** reports grants from Aimmune Therapeutics, a Nestlé Health Science company, during the conduct of the study; personal fees from DBV Technologies; grants and personal fees from FARE; and personal fees from ALK outside the submitted work. **Julie Wang** reports research support to her institution from Aimmune Therapeutics, a Nestlé Health Science company, DBV Technologies, Genentech, and Regeneron; support for attending Joint Task Force meetings for ACAAI; payments received from ALK‐Abello for participation on a data safety monitoring board; payments received from Jubilant HollisterStier for participation on an advisory board; and unpaid AAP Chair of the Executive Committee of the Section on Allergy and Immunology, as well as unpaid AAAAI Chair of the Anaphylaxis Dermatitis Drug Allergy Interest Section.

## Supporting information

Supporting Information S1
