# Supplementary material for: Children and caregiver proxy quality of life from peanut oral immunotherapy trials
Source: Clin Transl Allergy. 2022 Dec 19;12(12):e12213. doi: 10.1002/clt2.12213 (PMC9762119; doi:10.1002/clt2.12213)
Supplement: Supplementary file 1 — Supporting Information S1 [file CLT2-12-e12213-s001.docx]

**SUPPORTING INFORMATION**

**Supplemental Text**

Given the heterogeneity of the study design, such as differences in treatment duration or knowledge of treatment allocation (blinded vs open-label), combining results across trials was limited. For each domain, average score was considered the arithmetic average of the non-missing items in the domain. Total score was calculated as the average of the domain averages. For the FAQLQ version completed by the caregiver proxy for children aged 4-6 years, items that were completed but were not included for this age group were not counted toward the scoring as they should not have been completed originally. If less than 80% of the questions on the HRQoL instrument were answered, total scores for that version were not calculated; missing values were not imputed. During the development and validation of the FAQLQ for children, a response rate of ≥73% was obtained.^1^ HRQoL was assessed by longitudinal change in FAQLQ and FAIM scores and via responder analysis.

The data on quality of life collected in the studies in this analysis (PALISADE, ARTEMIS, RAMSES, ARC004, and ARC011) have been collected and analyzed using the specific scales that were applied in each individual study. These scales differ for individual parameters in terms of the characteristics used, so that it has not been possible to directly combine and interpret the results in the context of all the studies. In order to achieve the desired cross-study overview, it is necessary to reverse the scales in individual studies and thus harmonize the end values of the scales. This avoids inconsistencies and for the first time it is possible to interpret the individual results in the context of all studies and to pool the individual data. This also makes it possible to show that the results of the different studies are equivalent and that the statements on quality of life are consistent across studies.

**Reference**

1. Flokstra-de Blok BM, DunnGalvin A, Vlieg-Boerstra BJ, et al. Development and validation of a self-administered Food Allergy Quality of Life Questionnaire for children. *Clin Exp Allergy*. Jan 2009;39(1):127-37. doi:10.1111/j.1365-2222.2008.03120.x

**Table S1. Baseline Patient Demographics and Clinical Characteristics of Participants in PALISADE, ARTEMIS, and RAMSES**

| Parameter | ARC003  (PALISADE^†^) | | ARC010  (ARTEMIS^‡^) | | ARC007  (RAMSES^§^) | |
| --- | --- | --- | --- | --- | --- | --- |
|  | PTAH  n=372 | Placebo  n=124 | PTAH  n=132 | Placebo  n=43 | PTAH  n=337 | Placebo  n=168 |
| Male sex, n (%) | 208 (56) | 76 (61) | 68 (52) | 27 (63) | 218 (65) | 102 (61) |
| Peanut skin prick test  Mean wheal diameter, mm | 11 | 12 | 9.50 | 9.75 | 13.50 | 13.50 |
| Peanut-specific IgE  Median, kUA/L | 69 | 75 | 43.50 | 69.70 | 97.30 | 81.50 |
| History of peanut anaphylactic reactions,^¶^ n (%) | 269 (72) | 89 (72) | 58 (44) | 22 (51) | 204 (61) | 107 (64) |
| History of asthma, n (%) | 198 (53) | 65 (52) | 56 (42) | 14 (33) | 176 (52) | 77 (46) |
| Food allergies other than peanut, n (%) | 245 (66) | 80 (65) | 81 (61) | 21 (49) | 219 (65) | 90 (54) |

^†^Adapted from data in Table 1 of the PALISADE trial primary publication (From *The New England Journal of Medicine*, PALISADE Group of Clinical Investigators, AR101 Oral Immunotherapy for Peanut Allergy, 379, 1991-2001. Copyright © 2018 Massachusetts Medical Society. Reprinted with permission.)

^‡^Adapted from data in Table 1 of the ARTEMIS trial primary publication (Hourihane JO, et al. *Lancet Child Adolesc Health*. 2020;4[10]:728-39).

^§^Unpublished data; publication pending.

^¶^Represents lifetime history of systemic allergic reactions to peanut; severity of prior systemic allergic reactions was not collected.

**Table S2. Change in Scores on Domains of the FAQLQ**

| **Domain** | **PALISADE** | **ARC004 (1)** | **ARC004 (3A)** | **ARTEMIS** | **RAMSES** | **ARC011** |
| --- | --- | --- | --- | --- | --- | --- |
| Caregiver proxy-report: 4-6 years | | | | | | |
| Social and dietary limitations | | | | | | |
| Between treatment groups^†^ | 0.01 (-0.64, 0.67)  *P=*0.97 | NA | NA | -0.30 (-1.22, 0.62)  *P*=0.52 | -0.31 (-0.76, 0.15)  *P*=0.18 | NA |
| Within active treatment^‡^ | 0.143 (-0.18, 0.47) | -0.12 (-0.87, 0.62) | 0.76 (-0.51, 2.03) | -0.32 (-0.72, 0.08) | -0.35 (-0.63, -0.07) | -0.79 (-1.10, -0.47) |
| Emotional impact | | | | | | |
| Between treatment^†^ groups | -0.13 (-0.69, 0.43)  *P=*0.63 | NA | NA | -0.35 (-1.21, 0.50)  *P=*0.41 | -0.07 (-0.50, 0.35)  *P=*0.74 | NA |
| Within active treatment^‡^ | 0.31 (0.03, 0.58) | 0.30 (-0.51, 1.11) | 1.08 (-0.07, 2.23) | -0.01 (-0.38, 0.36) | -0.18 (-0.45, 0.08) | -0.26 (-0.61, 0.10) |
| Food anxiety | | | | | | |
| Between treatment^†^ groups | -0.38 (-1.00, 0.25)  *P=*0.24 | NA | NA | -0.18 (-1.25, 0.90)  *P=*0.74 | -0.32 (-0.84, 0.20)  *P=*0.23 | NA |
| Within active treatment^‡^ | 0.33 (0.02, 0.64) | 0.59 (-0.20, 1.37) | 0.91 (-0.36, 2.19) | -0.29 (-0.76, 0.18) | -0.30 (-0.63, 0.03) | -0.43 (-0.83, -0.03) |
| Caregiver proxy-report: 7-12 years | | | | | | |
| Social and dietary limitations | | | | | | |
| Between treatment^†^ groups | 0.04 (-0.35, 0.43)  *P=*0.85 | NA | NA | -0.54 (-1.19, 0.12)  *P=*0.11 | -0.08 (-0.40, 0.25)  *P=*0.65 | NA |
| Within active treatment^‡^ | -0.17 (-0.39, 0.06) | -0.65 (-1.02, -0.27) | -0.04 (-1.18, 1.10) | -0.25 (-0.69, -0.01) | -0.27 (-0.47, -0.07) | -0.73 (-0.99, -0.47) |
| Emotional impact | | | | | | |
| Between treatment^†^ groups | 0.07 (-0.30, 0.45)  *P=*0.70 | NA | NA | 0.18 (-0.54, 0.90)  *P=*0.62 | <0.01 (-0.29, 0.30)  *P=*0.99 | NA |
| Within active treatment^‡^ | -0.16 (-0.34, 0.06) | -0.32 (-0.65, 0.02) | 0.02 (-0.73, 0.77) | -0.10 (-0.47, 0.28) | -0.12 (-0.30, 0.06) | -0.47 (-0.71, -0.24) |
| Food anxiety | | | | | | |
| Between treatment^†^ groups | 0.14 (-0.26, 0.53)  *P=*0.49 | NA | NA | -0.38 (-1.20, 0.44)  *P=*0.36 | 0.10 (-0.24, 0.44)  *P=*0.58 | NA |
| Within active treatment^‡^ | -0.19 (-0.41, 0.04) | -0.43 (-0.77, -0.08) | -0.27 (-1.25, 0.71) | -0.42 (-0.85, <0.01) | -0.17 (-0.38, 0.04) | -0.69 (-0.96, -0.41) |
| Caregiver proxy-report: 13-17 years | | | | | | |
| Dietary restrictions | | | | | | |
| Between treatment^†^ groups | -0.27 (-0.69, 0.15)  *P*=0.20 | NA | NA | NA | NA | NA |
| Within active treatment^‡^ | -0.02 (-0.24, 0.19) | NA | NA | NA | NA | NA |
| Emotional impact | | | | | | |
| Between treatment^†^ groups | **-0.62 (-1.18, -0.07)**  ***P=*0.027** | NA | NA | **-0.69 (-1.35, -0.03)**  ***P=*0.04** | 0.27 (-0.10, 0.64)  *P=*0.15 | NA |
| Within active treatment^‡^ | -0.29 (-0.57, <-0.01) | -0.62 (-1.02, -0.22) | -0.90 (-1.84, 0.05) | -0.68 (-1.10, -0.26) | -0.09 (-0.31, 0.12) |  |
| Social restrictions | | | | | | |
| Between treatment^†^ groups | -0.25 (-0.91, 0.40)  *P=*0.45 | NA | NA | NA | NA | NA |
| Within active treatment^‡^ | -0.12 (-0.45, 0.22) | NA | NA | NA | NA | NA |
| Social and dietary limitations | | | | | | |
| Between treatment^†^ groups | NA | NA | NA | -0.07 (-0.63, 0.48)  *P=*0.79 | 0.12 (-0.23, 0.46)  *P=*0.50 | NA |
| Within active treatment^‡^ | NA | -0.48 (-0.84, -0.12) | -1.05 (-1.43, -0.67) | -0.29 (-0.64, -0.07) | -0.21, (-0.42, <-0.01) | -0.40 (-0.65, -0.15) |
| Food anxiety | | | | | | |
| Between treatment^†^ groups | -0.19 (-0.73, 0.35)  *P=*0.48 | NA | NA | -0.34 (-1.12, 0.44)  *P=*0.38 | 0.12 (-0.28, 0.53)  *P=*0.55 | NA |
| Within active treatment^‡^ | -0.08 (-0.36, 0.19) | -0.33 (-0.79, 0.12) | -1.42 (-1.94, -0.89) | -0.52 (-1.02, -0.02) | -0.13 (-0.37, 0.11) | -0.36 (-0.69, -0.02) |
| Self-report: 8-12 years | | | | | | |
| Allergy avoidance | | | | | | |
| Between treatment^†^ groups | -0.02 (-0.53, 0.49)  *P*=0.94 | NA | NA | NA | NA | NA |
| Within active treatment^‡^ | -0.37 (-0.65, -0.08) | NA | NA | NA | NA | NA |
| Allergy avoidance and dietary restrictions | | | | | | |
| Between treatment^†^ groups | NA | NA | NA | **-1.46 (-2.28, -0.65)**  ***P*<0.01** | 0.38 (-0.07, 0.84)  *P=*0.10 | NA |
| Within active treatment^‡^ | NA | -0.61 (-1.12, -0.09) | -0.54 (-1.60, 0.52) | -0.49 (-0.95, -0.02) | -0.33 (-0.61, -0.04) | -0.70 (-1.01, -0.39) |
| Emotional impact | | | | | | |
| Between treatment^†^ groups | -0.42 (-0.90, 0.05)  *P=*0.08 | NA | NA | -0.77 (-1.75, 0.22)  *P=*0.12 | 0.29 (-0.16, 0.74)  *P=*0.20 | NA |
| Within active treatment^‡^ | -0.61 (-0.87, -0.34) | -0.55 (-1.23, 0.13) | -1.00 (-2.73, 0.73) | -0.77 (-1.33, -0.20) | -0.64 (-0.92, -0.36) | -1.09 (-1.37, -0.81) |
| Risk of accidental exposure | | | | | | |
| Between treatment^†^ groups | -0.27 (-0.76, 0.23)  *P=*0.29 | NA | NA | **-1.44 (-2.49, -0.38)**  ***P*<0.01** | **0.63 (0.16, 1.10)**  ***P*<0.01** | NA |
| Within active treatment^‡^ | -0.43 (-0.71, -0.15) | -0.66 (-1.17, -0.14) | -0.05 (-1.33, 1.23) | -0.81 (-1.41, -0.20) | -0.23 (-0.52, 0.06) | -0.75 (-1.07, -0.42) |
| Dietary restrictions | | | | | | |
| Between treatment^†^ groups | -0.08 (-0.54, 0.37)  *P=*0.72 | NA | NA | NA | NA | NA |
| Within active treatment^‡^ | -0.29 (-0.54, -0.03) | NA | NA | NA | NA | NA |
| Self-report: 13-17 years | | | | | | |
| Allergy avoidance and dietary restrictions | | | | | | |
| Between treatment^†^ groups | <0.01 (-0.52, 0.52)  *P=*1.00 | NA | NA | -0.15 (-0.97, 0.67)  *P=*0.71 | 0.38 (-0.12, 0.88)  *P=*0.13 | NA |
| Within active treatment^‡^ | -0.31 (-0.57, -0.05) | -0.36 (-0.93, 0.21) | -0.85 (-1.60, -0.10) | -0.58 (-1.09, -0.06) | -0.43 (-0.73, -0.14) | -0.81 (-1.23, -0.38) |
| Emotional impact | | | | | | |
| Between treatment^†^ groups | -0.18 (-0.69, 0.33)  *P=*0.48 | NA | NA | -0.38 (-1.02, 0.26)  *P=*0.24 | 0.18 (-0.27, 0.63)  *P=*0.42 | NA |
| Within active treatment^‡^ | -0.68 (-0.94, -0.42) | -0.64 (-1.20, -0.08) | -1.10 (-1.82, -0.37) | -0.55 (-0.95, -0.15) | -0.43 (-0.70, -0.16) | -0.79 (-1.17, -0.40) |
| Risk of accidental exposure | | | | | | |
| Between treatment^†^ groups | -0.23 (-0.82, 0.35)  *P=*0.43 | NA | NA | -0.33 (-1.03, 0.37)  *P=*0.35 | -0.23 (-0.82, 0.35)  *P=*0.43 | NA |
| Within active treatment^‡^ | -0.37 (-0.67, -0.07) | -0.69 (-1.39, 0.02) | -0.94 (-2.13, 0.24) | -0.48 (-0.92, -0.04) | -0.37 (-0.67, -0.07) | -0.62 (-1.08, -0.16) |

Table cells with bolded text indicate results with statistically significant (*P*<0.05) values.

^†^Comparing differences in assessment scores between treatment groups (PTAH vs placebo) in placebo-controlled trials. Negative differences indicate PTAH is favored.

^‡^Comparing changes in assessment scores within the PTAH treatment group from baseline values. Negative differences indicate improvement in score.

**Abbreviations**: FAQLQ, Food Allergy Quality of Life Questionnaire; NA, not applicable; PTAH, peanut (*Arachis hypogaea*) allergen powder-dnfp.

**Table S3. Change in Scores on Domains of the FAIM**

| **Domain** | **PALISADE** | **ARC004 (1)** | **ARC004 (3A)** | **ARTEMIS** | **RAMSES** | **ARC011** |
| --- | --- | --- | --- | --- | --- | --- |
| Caregiver proxy-report: 4-12 years | | | | | | |
| Accidentally eat | | | | | | |
| Between treatment groups^†^ | -0.11 (-0.52, 0.30); *P=*0.59 | NA | NA | -0.51 (-1.31, 0.29); *P=*0.21 | **-0.61 (-0.90, -0.12); *P=*0.01** | NA |
| Within active treatment^‡^ | -0.16 (-0.38, 0.06) | -0.24 (-0.71, 0.24) | -0.24 (-1.05, 0.58) | -0.45 (-0.85, -0.05) | -0.56 (-0.80, -0.31) | -0.61 (-0.88, -0.35) |
| Severe reaction | | | | | | |
| Between treatment groups^†^ | **-0.61 (-1.04, -0.17); *P*<0.01** | NA | NA | -0.84 (-1.68, >-0.01); *P=*0.05 | **-0.82 (-1.21, 0.43); *P*<0.01** | NA |
| Within active treatment^‡^ | -0.69 (-0.92 -0.46) | -1.38 (-1.86, -0.90) | -2.06 (-2.65, -1.47) | -1.14 (-1.56, -0.72) | -0.91 (-1.15, -0.67) | -1.28 (-1.57, -0.10) |
| Die | | | | | | |
| Between treatment groups^†^ | -0.24 (-0.68, 0.20); *P=*0.29 | NA | NA | -0.11 (-1.08, 0.85); *P=*0.81 | **-0.46 (-0.87, -0.05); *P=*0.03** | NA |
| Within active treatment^‡^ | -0.44 (-0.68, -0.21) | -0.75 (-1.14, -0.35) | -1.47 (-2.10, -0.84) | -0.41 (-0.90, 0.08) | -0.72 (-0.97, -0.47) | -1.08 (-1.35, -0.80) |
| Do the right things | | | | | | |
| Between treatment groups^†^ | <0.01 (-0.48, 0.48); *P=*0.99 | NA | NA | 0.63 (-0.50, 1.75); *P=*0.27 | 0.30 (-0.14, 0.75); *P=*0.18 | NA |
| Within active treatment^‡^ | 0.03 (-0.22, 0.29) | 0.51 (0.03, 0.99) | -0.35 (-1.24, 0.54) | -0.02 (-0.58, 0.54) | 0.26 (-0.02, 0.53) | 0.15 (-0.22, 0.51) |
| Child thinks: accidental eat | | | | | | |
| Between treatment groups^†^ | -0.03 (-0.48, 0.42); *P=*0.90 | NA | NA | -0.83 (-1.93, 0.26); *P=*0.13 | -0.05 (-0.47, 0.37); *P=*0.81 | NA |
| Within active treatment^‡^ | -0.16 (-0.40, 0.08) | -0.20 (-0.69, 0.29) | 0.00 (-0.84, 0.84) | -0.48 (-1.03, 0.07) | -0.27 (-0.53, -0.01) | -0.50 (-0.78, -0.22) |
| Child thinks: severe reaction | | | | | | |
| Between treatment groups^†^ | **-0.71 (1.20, -0.21); *P*<0.01** | NA | NA | -0.87 (-1.97, 0.22); *P=*0.12 | -0.45 (-0.90, <0.01); *P=*0.05 | NA |
| Within active treatment^‡^ | -0.56 (-0.82, -0.29) | -0.69 (-1.17, -0.22) | -2.06 (-2.87, -1.25) | -0.58 (-1.13, -0.03) | -0.58 (-0.56, -0.30) | -1.08 (1.43, -0.74) |
| Child thinks: die | | | | | | |
| Between treatment groups^†^ | -0.19 (-0.70, 0.32); *P=*0.47 | NA | NA | >-0.01 (1.21, 1.20) *P=*0.99 | -0.43 (-0.90, 0.05); *P=*0.08 | NA |
| Within active treatment^‡^ | -0.25 (-0.52, 0.02) | -0.49 (-0.92, -0.06) | -1.50 (-2.48, -0.52) | -0.24 (-0.85, 0.37) | -0.35 (-0.64, -0.06) | -0.74 (-1.08, -0.40) |
| Child thinks: do the right things | | | | | | |
| Between treatment groups^†^ | -0.03 (-0.56, 0.50); *P=*0.91 | NA | NA | 0.19 (-0.98, 1.36); *P=*0.74 | 0.02 (-0.50, 0.54); *P=*0.93 | NA |
| Within active treatment^‡^ | -0.04 (-0.24, 0.33) | 0.40 (-0.14, 0.94) | 0.31 (-0.77, 1.40) | 0.22 (-0.81, 0.37) | 0.23 (-0.09, 0.55) | - |
| Caregiver proxy-report: 13-17 years | | | | | | |
| Accidentally eat | | | | | | |
| Between treatment groups^†^ | -0.20 (-0.97, 0.57); *P=*0.60 | NA | NA | 0.83 (-0.96, 1.13); *P=*0.87 | -0.20 (-0.97, 0.57); *P=*0.60 | NA |
| Within active treatment^‡^ | 0.06 (-0.33, 0.45) | 0.24 (-0.38, 0.85) | 0.00 (-2.23, 2.23) | -0.25 (-0.93, 0.43) | 0.06 (-0.33, 0.45) | -0.67 (-1.26, -0.05) |
| Severe reaction | | | | | | |
| Between treatment groups^†^ | -0.40 (-1.20, 0.40); *P=*0.33 | NA | NA | -0.86 (-2.62, 0.90); *P=*0.32 | -0.40 (-1.20, 0.40); *P=*0.33 | NA |
| Within active treatment^‡^ | -0.44 (-0.84, -0.04) | -2.41 (-3.50, -1.32) | -2.60 (-3.93, -1.27) | 1.42 (-2.57, -0.26) | 0.44 (-0.84, -0.04) | -0.66 (-1.26, -0.05) |
| Die | | | | | | |
| Between treatment groups^†^ | -0.64 (-1.42, 0.14); *P=*0.11 | NA | NA | **-1.44 (-2.72, -0.17); *P=*0.03** | -0.64 (-1.42, 0.14); *P=*0.11 | NA |
| Within active treatment^‡^ | -0.33 (-0.73, 0.06) | -1.82 (-2.54, -1.11) | -2.20 (-3.89, -0.51) | -1.00 (-1.83, -0.17) | -0.33 (-0.73, 0.06) | -0.66 (-1.26, -0.05) |
| Do the right things | | | | | | |
| Between treatment groups^†^ | -0.18 (-0.89, 0.54); *P=*0.63 | NA | NA | -0.23 (-0.78, 0.33); *P=*0.42 | -0.18 (-0.89, 0.54); *P=*0.63 | NA |
| Within active treatment^‡^ | -0.13 (-0.49, 0.23) | -0.06 (-0.72, 0.60) | 0.60 (-0.40, 1.60) | -0.02 (-0.35, 0.31) | -0.13 (-0.49, 0.23) | -0.66 (-1.26, -0.05) |
| Self-report: 8-12 years | | | | | | |
| Accidentally eat | | | | | | |
| Between treatment groups^†^ | -0.14 (-0.58, 0.29); *P=*0.52 | NA | NA | -0.44 (-1.53, 0.65); *P=*0.42 | 0.13 (-0.38, 0.63); *P=*0.62 | NA |
| Within active treatment^‡^ | -0.38 (-0.62, -0.14) | -0.29 (-0.68,0.10) | -0.56 (-1.22, 0.11) | -0.29 (-0.96, 0.39) | -0.23 (-0.54, 0.09) | - |
| Severe reaction | | | | | | |
| Between treatment groups^†^ | -0.64 (-1.36, 0.09); *P=*0.09 | NA | NA | -0.89 (-2.31, 0.52); *P=*0.21 | **-0.77 (-1.42,  -0.12); *P=*0.02** | NA |
| Within active treatment^‡^ | -0.89 (-1.30, -0.48) | -1.07 (-1.86, -0.27) | -2.56 (-3.87, -1.25) | -1.05 (-1.92, -0.17) | -1.04 (-1.45, -0.64) | - |
| Die | | | | | | |
| Between treatment groups^†^ | **-0.91 (-1.48, -0.35); *P*<0.01** | NA | NA | -1.07 (-2.72, 0.59); *P=*0.20 | 0.19 (-0.47, 0.85); *P=*0.56 | NA |
| Within active treatment^‡^ | -0.87 (-1.19, -0.56) | -0.67 (-1.44, 0.10) | -1.00 (-1.57, -0.43) | -1.14 (-2.17, -0.12) | -0.60 (-1.01, -0.19) | - |
| Do the right things | | | | | | |
| Between treatment groups^†^ | -0.10 (-0.74, 0.54); *P=*0.76 | NA | NA | 0.07 (-1.42, 1.56); *P=*0.93 | -0.07 (-0.68, 0.54); *P=*0.82 | NA |
| Within active treatment^‡^ | -0.32 (-0.67, 0.04) | -0.52 (-1.15, 0.12) | 0.33 (-1.01, 1.68) | 0.14 (-0.78, 1.06) | -0.36 (-0.74, 0.02) | - |
| Unable to eat foods | | | | | | |
| Between treatment groups^†^ | -0.10 (-0.65, 0.45); *P=*0.73 | NA | NA | -0.72 (-1.84, 0.40); *P=*0.20 | 0.49 (0.02, 0.95); *P=*0.39 | NA |
| Within active treatment^‡^ | -0.02 (-0.33, 0.29) | -0.10 (-0.62, 0.43) | -0.67 (-1.40, 0.06) | -0.33 (-1.03, 0.36) | -0.06 (-0.35, 0.23) | - |
| Affect things you do | | | | | | |
| Between treatment groups^†^ | 0.50 (-0.12, 1.11); *P=*0.12 | NA | NA | -0.43 (-1.49, 0.63); *P=*0.41 | 0.14 (-0.31, 0.59); *P=*0.53 | NA |
| Within active treatment^‡^ | -0.19 (-0.54, 0.16) | -0.32 (-0.88, 0.23) | -0.89 (-2.44, 0.66) | -0.43 (-1.08, 0.22) | 0.03 (-0.25, 0.31) | - |
| Expected outcome | | | | | | |
| Between treatment groups^†^ | NA | NA | NA | NA | NA | NA |
| Within active treatment^‡^ | NA | -0.64 (-1.07, -0.21) | -0.94 (-1.51, -0.38) | NA | NA | NA |
| Product avoidance | | | | | | |
| Between treatment groups^†^ | NA | NA | NA | NA | NA | NA |
| Within active treatment^‡^ | NA | -0.10 (-0.62, 0.43) | -0.67 (-1.40, 0.06) | NA | NA | NA |
| Social impact | | | | | | |
| Between treatment groups^†^ | NA | NA | NA | NA | NA | NA |
| Within active treatment^‡^ | NA | -0.32 (-0.88, 0.23) | -0.89 (-2.44, 0.66) | NA | NA | NA |
| Self-report: 13-17 years | | | | | | |
| Accidentally eat | | | | | | |
| Between treatment groups^†^ | -0.46 (-1.06, 0.14); *P=*0.13 | NA | NA | 0.07 (-0.87, 1.02); *P=*0.88 | -0.018 (-0.58, 0.55); *P=*0.95 | NA |
| Within active treatment^‡^ | -0.61 (-0.93, -0.30) | -0.77 (-1.26, -0.29) | -0.40 (-1.18, 0.38) | 0.07 (-0.50, 0.64) | -0.08 (-0.41, 0.24) | - |
| Severe reaction | | | | | | |
| Between treatment groups^†^ | -0.32 (-1.11, 0.48); *P=*0.43 | NA | NA | **-1.57 (-2.83, -0.32); *P=*0.02** | 0.31 (-1.02, 0.41); *P=*0.40 | NA |
| Within active treatment^‡^ | -0.70 (-1.11, -0.29) | -1.00 (-1.77, -0.23) | -1.60 (-3.62, 0.42) | -1.07 (-1.83, -0.31) | -0.41 (-0.82, <0.01) | - |
| Die | | | | | | |
| Between treatment groups^†^ | -0.07 (-0.67, 0.82); *P=*0.85 | NA | NA | **-1.29 (-2.31, -0.27); *P=*0.02** | 0.03 (-0.63, 0.68); *P=*0.94 | NA |
| Within active treatment^‡^ | -0.54 (-0.93, -0.16) | -1.09 (-1.83, -0.35) | -1.60 (-2.60, -0.60) | -0.79 (-1.40, -0.17) | -0.41 (-0.79, 0.03) | - |
| Do the right things | | | | | | |
| Between treatment groups^†^ | -0.16 (-0.81, 0.49); *P=*0.63 | NA | NA | -0.70 (-2.10, 0.71); *P=*0.31 | -0.12 (-0.71, 0.47); *P=*0.68 | NA |
| Within active treatment^‡^ | -0.66 (-0.99, -0.32) | -0.14 (-0.69, 0.42) | -0.80 (-1.53, 0.07) | -0.57 (-1.42, 0.28) | -0.22 (-0.56, 0.12) | - |
| Unable to eat foods | | | | | | |
| Between treatment groups^†^ | -0.29 (-0.68, 0.11); *P=*0.15 | NA | NA | -0.34 (-1.25, 0.57); *P=*0.44 | 0.13 (-0.30, 0.56); *P=*0.56 | NA |
| Within active treatment^‡^ | -0.29 (-0.49, -0.08) | -0.86 (-1.30, -0.43) | -0.60 (-1.08, -0.12) | -0.21 (-0.76, 0.33) | -0.31 (-0.55, -0.06) | - |
| Affect things you do | | | | | | |
| Between treatment groups^†^ | -0.10 (-0.80, 0.60); *P=*0.77 | NA | NA | -0.45 (-1.42, 0.53); *P=*0.35 | 0.03 (-0.54, 0.60); *P=*0.92 | NA |
| Within active treatment^‡^ | -0.26 (-0.62, 0.11) | -0.46 (-1.03, 0.12) | -1.00 (-1.88, -0.12) | -0.57 (-1.16, 0.02) | -0.27 (-0.60, 0.06) | - |
| Expected outcome | | | | | | |
| Between treatment groups^†^ | NA | NA | NA | NA | NA | NA |
| Within active treatment^‡^ | NA | -0.75 (-1.14, -0.36) | -1.10 (-2.12, -0.08) | NA | NA | NA |
| Product avoidance | | | | | | |
| Between treatment groups^†^ | NA | NA | NA | NA | NA | NA |
| Within active treatment^‡^ | NA | -0.86 (-1.30, -0.43) | -0.60 (-1.08, -0.12) | NA | NA | NA |
| Social impact | | | | | | |
| Between treatment groups^†^ | NA | NA | NA | NA | NA | NA |
| Within active treatment^‡^ | NA | -0.46 (-1.03, 0.12) | -1.00 (-1.88, -0.12) | NA | NA | NA |

Table cells with bolded text indicate results with statistically significant (*P*<0.05) values.

^†^Comparing differences in assessment scores between treatment groups (PTAH vs placebo) in placebo-controlled trials. Negative differences indicate PTAH is favored.

^‡^Comparing changes in assessment scores within the PTAH treatment group from baseline values. Negative differences indicate improvement in score.

**Abbreviations**: FAIM, Food Allergy Independent Measure; NA, not applicable; PTAH, peanut (*Arachis hypogaea*) allergen powder-dnfp.
